# Mitochondrial homeostasis regulates definitive endoderm differentiation of human pluripotent stem cells

**DOI:** 10.1038/s41420-022-00867-z

**Published:** 2022-02-17

**Authors:** Jing Lv, Ying Yi, Yan Qi, Chenchao Yan, Wenwen Jin, Liming Meng, Donghui Zhang, Wei Jiang

**Affiliations:** 1 Department of Biological Repositories, Frontier Science Center for Immunology and Metabolism, Medical Research Institute, Zhongnan Hospital of Wuhan University, Wuhan University, Wuhan, 430071 China; 2 State Key Laboratory of Biocatalysis and Enzyme Engineering, School of Life Science, Hubei University, Wuhan, 430062 China; 3 Human Genetics Resource Preservation Center of Wuhan University, Wuhan, 430071 China; 4 Hubei Provincial Key Laboratory of Developmentally Originated Disease, Wuhan, 430071 China

**Keywords:** Stem-cell differentiation, Differentiation

## Abstract

Cellular organelles play fundamental roles in almost all cell behaviors. Mitochondria have been reported to be functionally linked to various biological processes, including reprogramming and pluripotency maintenance. However, very little about the role of mitochondria has been revealed in human early development and lineage specification. Here, we reported the characteristics and function of mitochondria during human definitive endoderm differentiation. Using a well-established differentiation system, we first investigated the change of mitochondrial morphology by comparing undifferentiated pluripotent stem cells, the intermediate mesendoderm cells, and differentiated endoderm cells, and found that mitochondria were gradually elongated and matured along differentiation. We further analyzed the expression pattern of mitochondria-related genes by RNA-seq, indicating that mitochondria became active during differentiation. Supporting this notion, the production of adenosine triphosphate (ATP) and reactive oxygen species (ROS) was increased as well. Functionally, we utilized chemicals and genome editing techniques, which could interfere with mitochondrial homeostasis, to determine the role of mitochondria in human endoderm differentiation. Treatment with mitochondrial inhibitors, or genetic depletion of mitochondrial transcription factor A (*TFAM*), significantly reduced the differentiation efficiency of definitive endoderm. In addition, the defect in endoderm differentiation due to dysfunctional mitochondria could be restored to some extent by the addition of ATP. Moreover, the clearance of excessive ROS due to dysfunctional mitochondria by N-acetylcysteine (NAC) improved the differentiation as well. We further found that ATP and NAC could partially replace the growth factor activin A for definitive endoderm differentiation. Our study illustrates the essential role of mitochondria during human endoderm differentiation through providing ATP and regulating ROS levels, which may provide new insight for metabolic regulation of cell fate determination.

## Introduction

Mitochondria are considered highly plastic organelles, which continuously alter their morphology by undergoing dynamic processes to respond to cellular demands [1]. Mitochondria are rare organelles with their own genome, some of which are required for the respiratory activity to supply the cell with metabolic energy [2]. Mitochondrial respiratory can impact cell proliferation through the NAD+/NADH ratio [3]. Furthermore, mitochondria also provide reaction sites for many catabolism and anabolism, such as the tricarboxylic acid (TCA) cycle, fatty acid oxidation, and certain phospholipid synthesis [4]. These metabolic processes not only provide reactive raw materials for energy production but also produce some metabolites involved in all aspects of cell activity. A few studies suggested that mitochondrial dysfunction led to impaired energy production and thus contributed to the regulation of various cell activities [5, 6]. Besides, the abnormal mitochondrial function can lead to many diseases, such as Parkinson’s disease, respiratory failure, liver failure, and diabetes mellitus [7]. Therefore, studies on the role of mitochondria in various biological processes and disease pathogenesis have attracted great attention.

Early embryo development is the basis and start of organogenesis, but the study of human early development is relatively hindered due to the limitation of materials and technologies. Pluripotent stem cells (PSCs) including embryonic stem cells (ESCs) and induced PSCs (iPSCs), have unique advantages that can self-renew indefinitely and have the ability to give rise to cells of all three embryonic germ-layers, providing an ideal platform to study cell fate determination and early lineage specification [8]. Beyond the well-studied transcriptional factors, signal pathways, and epigenetic modifications, more and more reports indicate metabolic remodeling including mitochondrial homeostasis plays an important role in cell fate determination as well [9]. Choi and colleagues reported that three types of mouse blastocyst-derived stem cells (i.e., ESCs, trophoblast stem cells, and extraembryonic endoderm cells) have their own characteristics on the mitochondrial dynamics and metabolic profile [10]; Li and colleagues described mitochondrial mass and activity were both regulated and activated by TGF-β signaling during endoderm differentiation [11]. These studies suggest a potential role of mitochondria in early lineage specification. In fact, several studies had illustrated abnormal mitochondrial function resulted in a loss of differentiation ability of stem cells. Hoque and colleagues reported that interference of mitochondrial homeostasis by the genetic deletion of the mitochondrial fission protein, Drp1, or pharmacological inhibition, could promote the differentiation of human PSCs into cardiac lineage accompanied by a metabolic shift from glycolysis towards oxidative phosphorylation [12]. Forni and colleagues reported impaired mitochondrial homeostasis by knocking down Mfn2 during adipogenesis and osteogenesis, or the overexpression of a dominant-negative form of Drp1 during chondrogenesis could destroy their differentiation respectively, through altering bioenergetic profiles [13]. In addition, siRNA-based knockdown of mitochondrial transcription factor A (TFAM), and hypoxia- or chemical-induced mitochondrial dysfunction can both suppress adipogenic differentiation of human mesenchymal stem cells [14]. Another study also showed that abnormal mitochondria caused by the deletion of gene *OPA1* or *MFN1/2* could impair the self-renewal and differentiation of neural stem cells through a reactive oxygen species (ROS)-mediated process [15]. One study pointed out that mitochondrial mass and activity have a decrease during the early nascent ectoderm differentiation [16], which are usually increased in somatic cells [17], indicating that mitochondrial metabolism may have distinct patterns during early differentiation.

Here, we aimed to investigate the role of mitochondria in the definitive endoderm (DE) differentiation of human ESCs/iPSCs. We checked the mitochondrial morphology and activity during endoderm differentiation, and then dissected the biological function through pharmacological inhibitors and a genetic approach to interfere with mitochondrial homeostasis. Finally, we analyzed whether the two main downstream outputs of mitochondrial activity, adenosine triphosphate (ATP) and ROS, contributed to endoderm differentiation.

## Results

### Mitochondria become more mature during DE differentiation

**Fig. 1:**
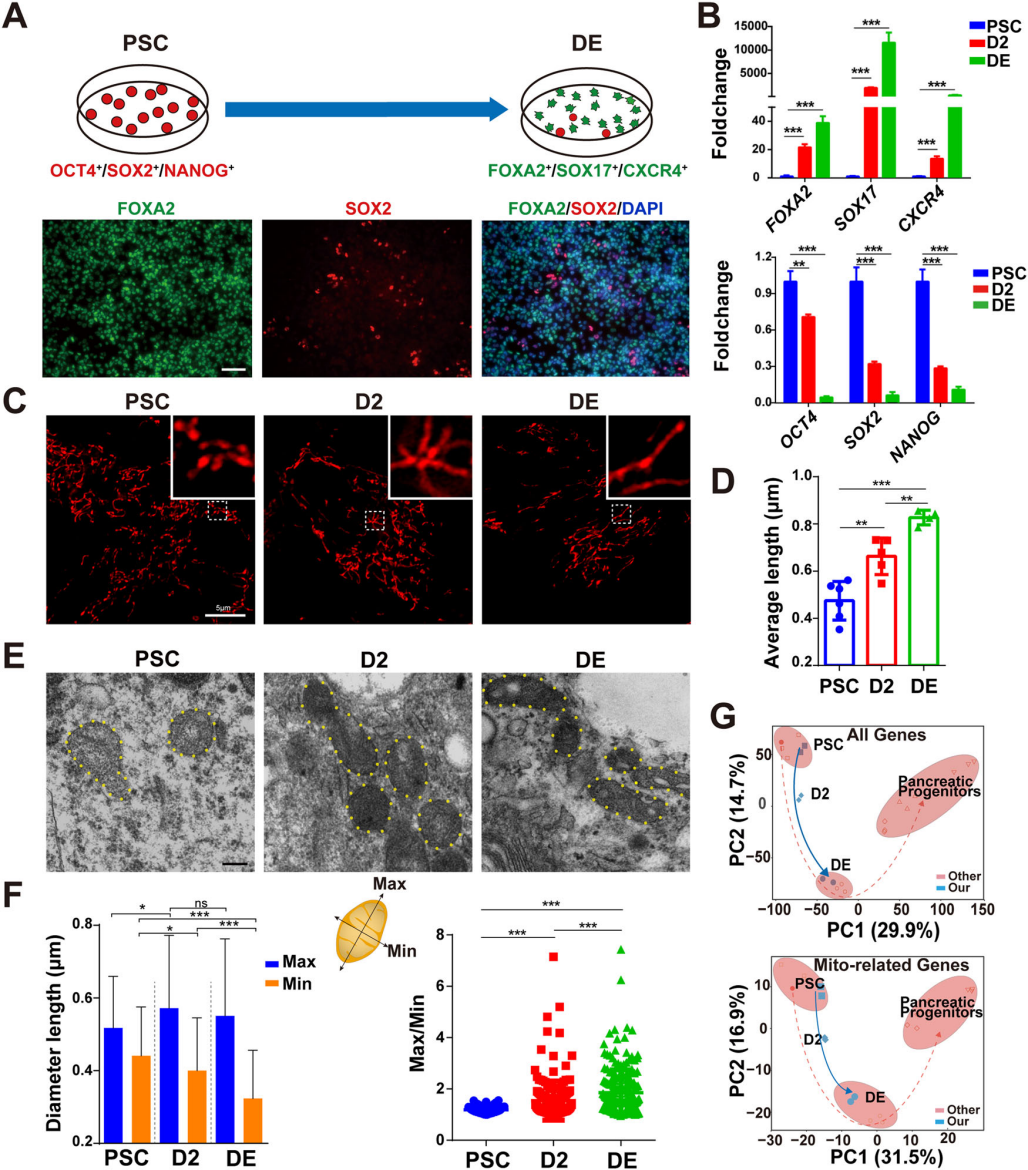
Mitochondria are more mature during human DE differentiation. **A** Scheme of DE differentiation. FOXA2^+^ (green), SOX17^+^, and CXCR4^+^ cells represented differentiated DE cells, and OCT4^+^, SOX2^+^ (red), and NANOG^+^ represented pluripotent cells (nucleus, DAPI, blue). Scale bar = 400 μm. **B** RNA expression analysis of differentiation markers and pluripotency markers during DE differentiation. Data are normalized to the mRNA level of PSCs (*n* = 3). **C** Representative images of MitoTracker staining observed through confocal in DE differentiation of iPSCs. Scale bar = 5 μm. **D** Quantitative statistics of mitochondrial average length in (**C**) using the MINA tool. **E** Representative mitochondrial images of ESCs, D2, DE were observed through a transmission electron microscope. Scale bar = 2 μm. **F** The ratio of Max/Min axes and diameter length of mitochondria (**E**) were statistically analyzed from about 20 independent images. **G** PCA analysis of all genes (top) and mitochondria-related genes (bottom) in pancreatic differentiation (GSE114099, red) and our DE differentiation (blue). All data are shown as mean ± SD. ns, not significant. *P < 0.05, **P < 0.01 and ***P < 0.001.

To understand the changes of mitochondria during DE differentiation, we first established an efficient and simple DE differentiation system from human ESC (HUES8) and iPSC (PGP1) mainly based on activin A treatment [18]. After 5 days’ differentiation, we could obtain significant FOXA2-positive cells from HUES8 through immunofluorescence (Fig. 1A), and the RNA levels of DE-related genes (*FOXA2*, *SOX17*, *CXCR4*) were dramatically increased along with the decreased expression of pluripotent genes (*OCT4*, *SOX2*, *NANOG*) (Fig. 1B), which was consistent with RNA sequencing (RNA-seq) data (Fig. S1A). Transcriptome analysis also indicated the successful DE differentiation, evidenced by the enrichment of terms such as the upregulated endoderm development (Fig. S1B), WNT signaling pathway, and TGF-beta signaling pathway (Fig. S1C). Next, we used MitoTracker staining to observe mitochondrial morphology at different stages: PSC, intermediate stage at differentiation day 2 (D2), DE. We observed the granular mitochondria became more tubular and formed net-like reticular networks during differentiation (Fig. 1C). To fully illustrate the relative change of mitochondrial length, we used a statistical method, mitochondrial network analysis, which can analyze the branched and complex mitochondrial networks [19]. The results showed that the average length of mitochondria had a significant increase in DE cells compared to undifferentiated iPSCs (Fig. 1D) or ESCs (Fig. S1D). The result based on transmission electron microscopy showed that the shape of mitochondria was gradually from globular (PSC) to elongated (D2, DE) (Fig. 1E) as differentiation advanced, consistent with the quantitative analysis [10, 20] of the relative ratio measurement of length (Max) and width (Min) of mitochondria (Fig. 1F).

In addition to the morphology, we carried out RNA high-throughput sequencing of DE differentiation to comprehensively understand the dynamic changes of mitochondria-related genes. By analyzing our homemade RNA-seq data and the recently published dataset about endodermal pancreatic lineage differentiation [21], the distribution of samples was highly similar based on either all genes or mitochondria-related genes only (Fig. 1G), suggesting significant mitochondrial remodeling during endodermal lineage differentiation. Of the 986 mitochondria-related genes, 185 were included in the ES/DE differentially expressed genes (DEGs) (Fig. S1E). More interestingly, most of these mitochondrial genes are upregulated in DE cells (Fig. S1F). Besides, the expression of mitochondrial homeostasis (biogenesis, fusion and fission)- related genes was dynamically changed (Fig. S1G).

### Mitochondria are more active in DE cells than PSCs

**Fig. 2:**
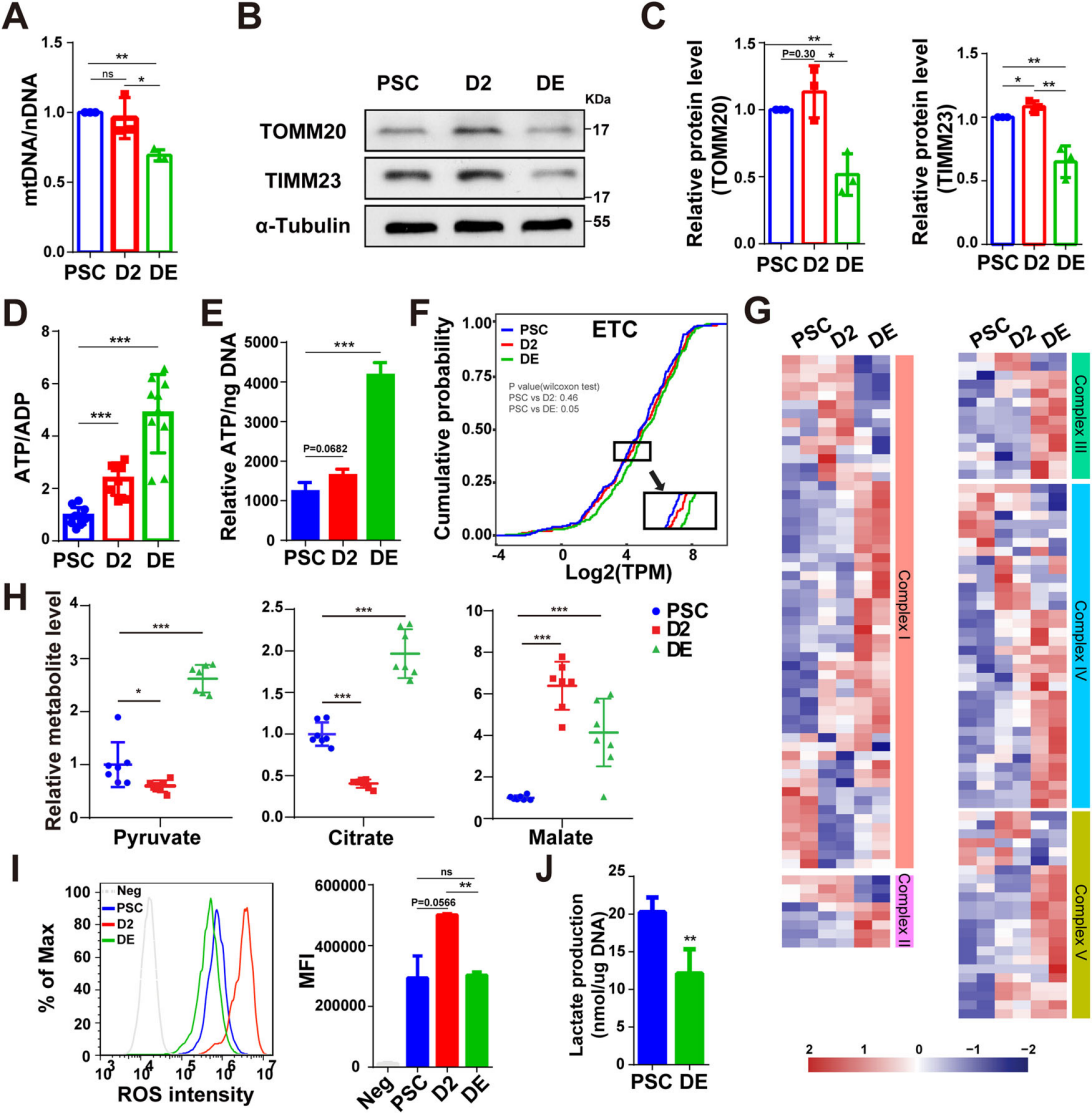
Mitochondria become more active along with DE differentiation. **A** The expression of mtDNA relative to the nuclear gene GAPDH in DE differentiation by RT-PCR (*n* = 3). **B** The western blot of TOMM20, TIMM23, and α-tubulin protein during DE differentiation. **C** Quantitative statistics of the TOMM20 and TIMM23 protein levels (tubulin as the reference protein), corresponding to (**B**) (*n* = 3). **D**, **E** Total intracellular ATP/ADP (**D**) (*n* = 10) and relative ATP content (normalized with DNA content) (**E**) of cells at three different stages along DE differentiation (*n* = 3). **F** Cumulative distribution curves of Log2(TPM) of ETC subunits-related genes (*n* = 124). The *P*-value was calculated by a Wilcoxon test. **G** Heatmap showing the expression levels of each complex related gene of ETC by RNA-seq. **H** Content of representative TCA metabolites in DE differentiation by untargeted metabolomics (normalized to PSCs) (*n* = 7). **I** The intracellular ROS level by FACS (left) and the corresponding mean fluorescence intensity (MFI) (right) in DE differentiation of ESCs (*n* = 3). Neg, negative control. **J** The assay of lactate production in PSCs and DE cells (*n* = 4). All data are shown as mean ± SD. ns, not significant. *P < 0.05, **P < 0.01 and ***P < 0.001.

To further examine mitochondrial metabolism as hPSCs exit pluripotency and differentiate into endoderm, we assessed mitochondrial activity during different stages of endoderm differentiation. The expression of both mitochondrial DNA (mtDNA) and mitochondrial marker proteins TOMM20 (translocase of outer mitochondrial membrane 20) and TIMM23 (translocase of inner mitochondrial membrane 23) was lower in DE cells than in PSCs (Fig. 2A–C), which indicated the number of mitochondria had a decrease after a burst as DE differentiation progressed, a similar phenomenon to the previous study in neural differentiation [16]. Mitochondria as the “powerhouse” provide most of ATP to cells through the process of OXPHOS on the electron transport chain (ETC) and substrate-level phosphorylation in the TCA cycle, and they exert crucial functions in ROS signaling as well [9, 22–24]. To investigate the mitochondrial activity, we first examined the total intracellular ATP/ADP ratio and ATP production and found both were increased after DE differentiation (about 5 and 4 times, respectively) (Fig. 2D, E). Next, we analyzed the mRNA expression of ETC-related genes with RNA-seq data, most of which had an elevated expression in the DE stage (Fig. 2F, G). Meanwhile, from untargeted metabolomics data, we found that the levels of several key metabolites (pyruvate, citrate, and malate) in the TCA cycle were eventually increased at the DE stage (Fig. 2H). In addition, we examined the intracellular ROS levels by FACS (Fig. 2I). The data showed that the ROS level was increased significantly after the initiation of endoderm differentiation; then the ROS level was rapidly decreased to a similar degree of PSCs. Similar results were also obtained in human iPSCs (Fig. S2A, B).

Given that the cytoplasmic glycolysis can also provide ATP for cells in addition to mitochondria, we examined the glycolysis level and found the lactate production (Fig. 2J) and extracellular acidification rate (ECAR) with Seahorse assay (Fig. S2C) became lower in DE cells, indicating a decreased glycolysis in DE cells compared to PSCs, consistent with previous studies [25, 26]. Combined with the above results, we speculated that there was a higher energy production in the mitochondria in DE cells. Surprisingly, we did not observe a significantly higher level of oxygen consumption rate (OCR) in DE cells (Fig. S2D). Similar results have also emerged in the other reports [27]. We noticed that in addition to glycolysis and OXPHOS, mitochondrial substrate-level phosphorylation (mSLP) can also generate sufficient ATP to meet cellular demands in the presence or absence of oxygen [22, 23]. Indeed, we observed the expression of several members of the SUCL (succinate-CoA ligase) family (*SUCLG1*, *SUCLG2*, *SUCLA2*), the key enzyme of mSLP, was obviously upregulated in DE cells compared to PSCs (Fig. S2E). Nevertheless, these data suggested that the total mitochondrial activity was likely higher in DE cells compared to PSCs.

### Genetic targeting mitochondria by TFAM knockout impairs DE differentiation

**Fig. 3:**
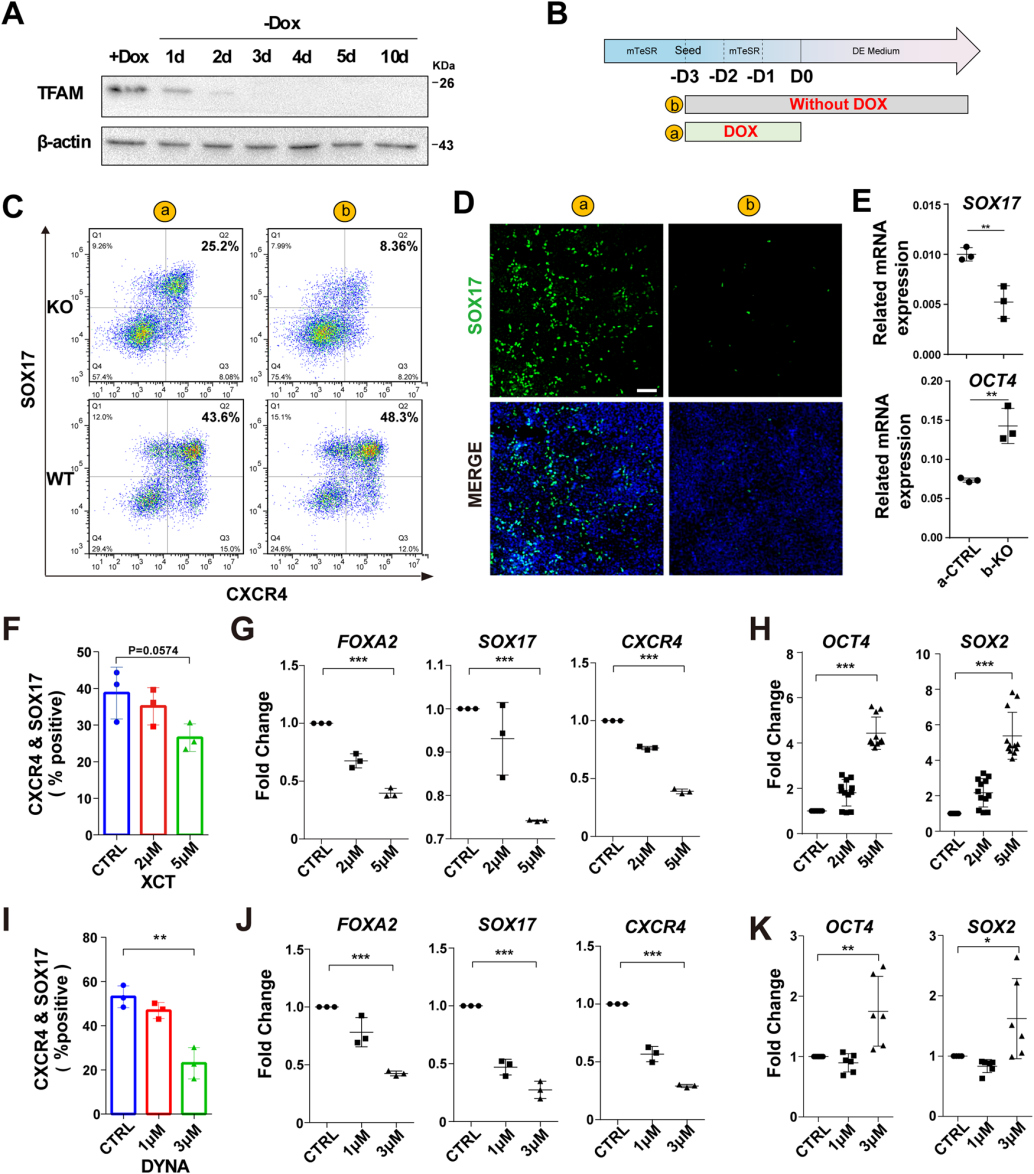
Interference with mitochondrial homeostasis impairs DE differentiation. **A** The western blot of TFAM and β-actin protein along with the removal time of doxycycline. **B** Schematic illustration of the differentiation protocol for inducible TFAM knockout PSCs. **C** Flow cytometric analysis to determine the change in differentiation efficiency between induced knockout and wild-type iPSCs. SOX17^+^ and CXCR4^+^ cells represented differentiated DE cells. **D** Immunofluorescence analysis of DE cells marked with SOX17 (green) (nucleus, DAPI, blue). Scale bar = 200 μm. **E** mRNA expression of endoderm- or pluripotency-marker *SOX17*, *OCT4* in DE cells treated with or without 100 ng/ml doxycycline (*n* = 3). **F** Flow cytometric analysis to measure the percentage of cells expressing both differentiation markers (SOX17 and CXCR4) treated with XCT790 (XCT) in DE differentiation (*n* = 3). **G** mRNA expression of endoderm transcription factors *FOXA2*, *SOX17*, and *CXCR4* in DE cells treated with XCT (*n* = 3). **H** mRNA expression of pluripotent markers *OCT4* and *SOX2* in DE cells treated with XCT (*n* = 12). **I** Flow cytometric analysis to measure the percentage of cells expressing both DE markers (SOX17 and CXCR4) treated with dynasore (DYNA) (*n* = 3). **J** mRNA expression of endoderm transcription factors *FOXA2*, *SOX17*, and *CXCR4* in DE cells treated with dynasore (*n* = 3). **K** mRNA expression of pluripotent markers *OCT4* and *SOX2* in DE cells treated with dynasore (*n* = 6). The data of RT-qPCR are normalized to the mRNA level of untreated (control) DE cells. All data are shown as mean ± SD. *P < 0.05, **P < 0.01 and ***P < 0.001.

To further understand whether the normal mitochondrial function is required for DE differentiation, we first used a genetic approach to interfere with the function of mitochondria. Mitochondrial transcription factor A (TFAM), a nuclear-encoded protein, can bind mtDNA to maintain the mitochondrial genome and activate the transcription of genes encoded by the mitochondrial genome [28]. The low level of TFAM leads to mitochondrial dysfunction [29]. Therefore, we targeted *TFAM* to explore the effect of mitochondrial function [30] on DE differentiation. We targeted the exon 1 of TFAM by CRISPR/Cas9 system, and obtained only heterozygous TFAM (TFAM^+/^^−^) cells with a frameshift mutation, but not homozygous cells. Compared with wild-type PSCs, the expression of mtDNA of TFAM^+/−^ cells decreased approximately 80% (Fig. S3A). Meanwhile, the basal respiration and maximum respiration also had a significant reduction in TFAM^+/−^ cells with Seahorse assay (Fig. S3B). These results indicated effective targeting. Then we differentiated the wild-type and TFAM^+/−^ iPSCs for 5 days using the protocol described above. Immunofluorescence showed a decrease in the percentage of endoderm marker FOXA2 after differentiation compared to wild-type cells (Fig. S3C), which predicted that TFAM was required for endoderm differentiation. To better prove this view, we established an inducible exogenous TFAM expression system based on endogenous TFAM knockout, which is similar to the previous reports that knock out the essential gene *DICER* [31]. For this inducible knockout cell line, we found that 100 ng/mL doxycycline (Dox) could induce a similar exogenous protein expression level to endogenous TFAM in PSCs. Since the expression of TFAM protein almost disappeared after three days of doxycycline removal (Fig. 3A), we seeded the cells three days before starting differentiation to get rid of TFAM protein by withdrawing Dox (Fig. 3B). The flow cytometric results showed that the SOX17 and CXCR4 double-positive percentage without Dox treatment were about 1/3 of the group with TFAM expression (8.38% vs. 25.2%). We also confirmed the treatment of doxycycline alone had little effect on the differentiation of wild-type PSCs (Fig. 3C). The proportion of SOX17-positive cells examined by immunofluorescence (Fig. 3D) was consistent with the flow cytometric results. Furthermore, the mRNA expression of *SOX17* was significantly decreased by 50%, accompanied by an upregulation of *OCT4* expression, as compared to wild-type PSCs (Fig. 3E). These results suggest that TFAM is required when PSCs exit pluripotency for DE differentiation, indicating the important role of normal mitochondria.

### Chemicals targeting mitochondrial homeostasis block DE differentiation

Next, we explored whether targeting mitochondria by small chemicals can hinder DE differentiation as well. Since chemical XCT790 (XCT for short) acts as an ERRα (estrogen-related receptor α) inverse agonist and has been demonstrated to drastically interfere with mitochondrial biogenesis in cancer cells [32], illustrated in Fig. S4A, we tested the effect of XCT to assess the role of mitochondrial homeostasis in DE differentiation. The results showed the treatment of XCT significantly decreased the percentage of FOXA2-, SOX17-, and CXCR4-positive cells (about 20%) measured by flow cytometric analysis and immunofluorescence assay (Fig. 3F, Fig. S4B, C). RT-qPCR data showed that the RNA levels of *FOXA2*, *SOX17*, and *CXCR4* were significantly decreased along with the increased concentration up to 5 μM of XCT, which had little effect on cell survival (Fig. 3G). Meanwhile, there was an upregulation of *OCT4* and *SOX2* expression (pluripotent genes) (Fig. 3H). Hence these data demonstrate an important role of mitochondrial biogenesis in regulating DE differentiation.

In addition to mitochondrial biogenesis, mitochondrial homeostasis is also ensured by the fission process. Here we applied chemicals targeting mitochondrial fission to explore the effect of mitochondrial homeostasis on endodermal differentiation. First, we treated PSCs with dynasore, that interferes with the GTPase activity of DRP1 (also called DNM1L), a mitochondrial fission protein [33]. As shown in Fig. 3I and Fig. S4D, the differentiation efficiency of DE determined by double positive of CXCR4 and SOX17 was decreased more than half when treated with 3 μM dynasore. RNA analysis showed that the expression levels of *FOXA2*, *SOX17*, and *CXCR4* were also significantly decreased up to 50% in a concentration-dependent manner (Fig. 3J), with a certain degree of upregulation of pluripotent genes *OCT4* and *SOX2* (Fig. 3K). A similar result that dynasore treatment strongly repressed DE differentiation was also observed in iPSC line PGP1 (Fig. S4E). To further confirm the importance of mitochondrial homeostasis, we used Mdivi-1, another well-recognized inhibitor of DRP1 [32]. Treatment with 10 μM Mdivi-1 also resulted in a reduced percentage of CXCR4- and SOX17-double-positive cells (~20%) (Fig. S4F), and the cells were just normally healthy (Fig. S4G). These data indicate that both genetic interference and pharmacological inhibition of mitochondrial homeostasis can impair the DE differentiation from human PSCs.

### ATP contributes to DE differentiation caused by mitochondrial dysfunction

**Fig. 4:**
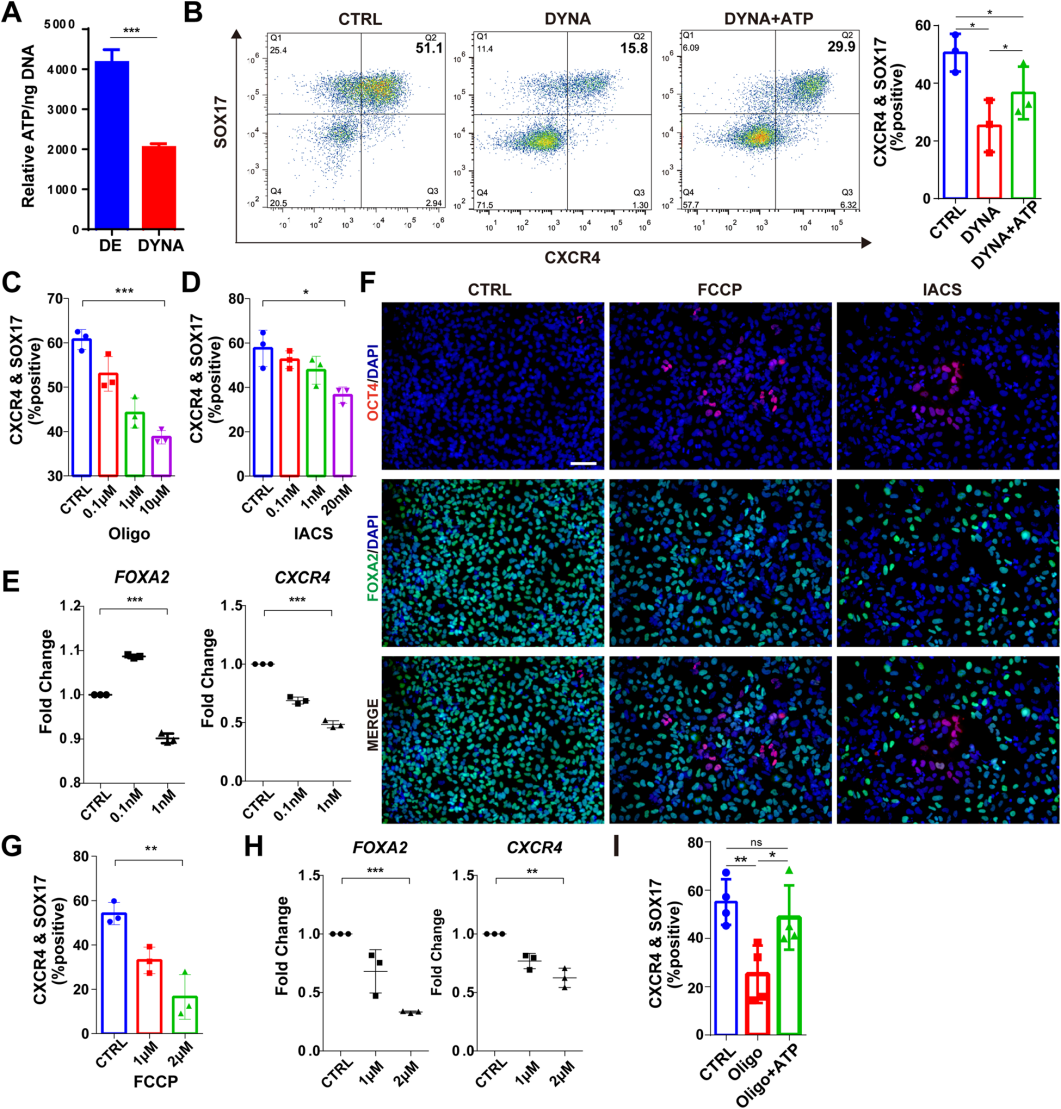
ATP is functionally involved in DE differentiation. **A** Total intracellular ATP content of DE cells treated with dynasore (*n* = 3). **B** Percentage of CXCR4 and SOX17-positive cells in DE differentiation treated with dynasore and 1 mM ATP measured by flow cytometry (*n* = 3). **C** Flow cytometry analysis to measure the percentage of CXCR4 and SOX17-positive cells in DE differentiation treated with oligomycin (oligo) (*n* = 3). **D** Flow cytometry analysis to measure the percentage of CXCR4 and SOX17-positive cells in DE differentiation treated with IACS (*n* = 3). **E** mRNA expression of endoderm factors FOXA2 and CXCR4 in DE cells treated with IACS (*n* = 3). **F** Immunofluorescence microscopy of definitive endoderm cells for FOXA2 (green) and OCT4 (red) treated with 2 μM FCCP or 20 nM IACS. DAPI, blue. Scale bar = 400 μm. **G** Flow cytometry analysis to measure the percentage of CXCR4 and SOX17-positive cells in DE differentiation treated with FCCP (*n* = 3). **H** mRNA expression of endoderm factors *FOXA2* and *CXCR4* in DE cells treated with FCCP (*n* = 3). **I** Flow cytometric analysis of CXCR4- and SOX17-positive cells in DE differentiation treated with 1 μM oligomycin plus 0.5 mM ATP (*n* = 4). The data of RT-PCR are normalized to the mRNA level of untreated (control) DE cells. All data are shown as mean ± SD. ns, not significant. *P < 0.05, **P < 0.01 and ***P < 0.001.

Mitochondria play multiple important functions in the cell, including metabolism (anabolism and catabolism), Ca^2+^ homeostasis, cellular signaling pathways. One of the main functions of mitochondria is the production of ATP for cellular energy needs [34, 35]. Therefore, we measured the total cellular ATP concentration in differentiated DE cells treated with or without the pharmacological mitochondrial inhibitor using the luminescent ATP detection assay. The result suggested the total cellular ATP concentration was reduced about half after 3 μM dynasore treatment (Fig. 4A). MitoTracker staining indicated that mitochondria in cells treated with dynasore or Mdivi-1 showed larger tubes compared with control (Fig. S5A). Recently, one group reported that DRP1-knockdown mouse embryonic fibroblasts showed mitochondria with abnormally swollen tubes due to the excessive fusion, which disrupted the normal mitochondrial network structure and caused impaired mitochondrial function [36], consistent with our observation of decreased ATP level upon dynasore treatment (Fig. 4A). Thus, disturbed mitochondrial homeostasis led to mitochondrial dysfunction. Next, we were wondering whether the changed ATP production contributes to the defective DE differentiation due to dysfunctional mitochondria, so we applied exogenous ATP during DE differentiation treated together with 3 μM dynasore [37]. Again, the treatment of dynasore caused a significant loss in DE markers SOX17 and CXCR4; however, this inhibitory effect of dynasore was partially reversed by the addition of 1 mM ATP (Fig. 4B), strongly indicated ATP production was important for mitochondria-mediated DE differentiation.

To avoid the non-specific effects of dynasore and further explore the mitochondrial function, we directly interfered with ATP synthesis and other proteins of ETC by chemical small molecules (Fig. S5B) to see if there is a similar phenotype caused by dysfunctional mitochondria. We first used oligomycin A, a direct known inhibitor of ATP synthase [38]. Treatment of human ESCs with different concentrations of oligomycin A during endoderm differentiation resulted in up to a 22% decrease in the percentage of positive DE cells (Fig. 4C). Then we used IACS-010759 (IACS for short), a chemical targeting the respiratory chain complex II that represents the connection point between the TCA and the ETC [39]. Treatment with IACS at 0.1–20 nM significantly decreased the percentage of CXCR4- and SOX17-positive DE cells (Fig. 4D), similar to the treatment with dynasore. Quantitative reverse transcription-polymerase chain reaction (RT-PCR) analysis confirmed the impaired endoderm differentiation of ESCs by IACS treatment, where expression levels of genes encoding endodermal factors *FOXA2* and *CXCR4* were significantly downregulated (Fig. 4E). A similar decrease in the percentage of endoderm cells was also observed in DE differentiation treated with carbonylcyanide-4-trifluoromethoxyphenylhydrazone (FCCP), the mitochondrial uncoupling agent [40]. 2 μM FCCP treatment led to a decrease in the percentage of CXCR4-positive and SOX17-positive cells in ESCs (from about 54.1 to 16.5%, Fig. 4F, G) and iPSCs (from about 43.5 to 27.6%, Fig. S5C). Analysis of RNA expression levels of endodermal markers showed significant downregulation of *FOXA2* and *CXCR4*, indicating reduced endoderm differentiation upon FCCP treatment (Fig. 4H). We also examined DE differentiation after ATP supplementation in cells with reduced ATP levels caused by oligomycin treatment, showing an obvious rescue of DE differentiation efficiency (Fig. 4I). These data together suggest that the ATP pathway is a critical downstream of mitochondrial function for DE differentiation.

### Increased ROS levels due to mitochondrial dysfunction contribute to impaired DE differentiation

**Fig. 5:**
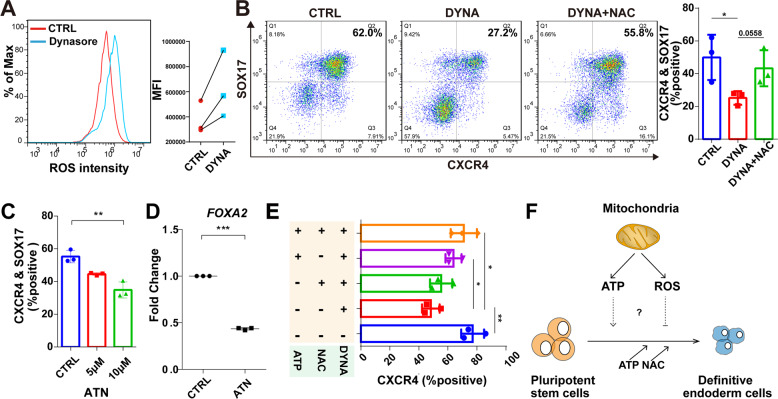
The clearance of ROS can rescue the impaired DE differentiation due to mitochondrial dysfunction. **A** Flow cytometric analysis to determine the changes of intracellular ROS level during DE differentiation treated with (DYNA) or without (CTRL) dynasore. The mean fluorescence intensity (MFI) of flow cytometry as shown on the right (*n* = 3). **B** Flow cytometry analysis of DE marker with the treatment of dynasore, and dynasore plus 3 mM NAC, respectively (*n* = 3). **C** Flow cytometry analysis of markers of DE cells when treated with ATN (*n* = 3). **D** The mRNA expression of *FOXA2* during DE differentiation treated with 10 μM ATN. Data are normalized to the mRNA level of untreated (control) DE cells (*n* = 3). **E** Percentage of CXCR4-positive cells (flow cytometry) in the endoderm differentiation treated with 3 μM dynasore, 1 mM NAC or 0.5 mM ATP (*n* = 3). **F** Schematic illustration of mitochondrial regulation to DE differentiation. All data are shown as mean ± SD. *P < 0.05, **P < 0.01 and ***P < 0.001.

The intracellular content of ROS has been frequently reported to be associated with mitochondrial imbalance [1, 35], so we next examined whether the inhibition of DE differentiation with chemical intervention is linked to ROS change. The flow cytometric analysis showed intracellular ROS levels were sharply increased in DE cells with pharmacological inhibitor treatment compared with the untreated group (Fig. 5A), consistent with previous reports that dysregulated mitochondrial homeostasis led to increased ROS levels [1, 41]. To determine whether the impaired DE differentiation is caused by the extra ROS, we treated with N-acetylcysteine (NAC), an inhibitor of ROS [42]. The result showed that the inhibitory differentiation by the treatment of mitochondrial inhibitor was rescued in part by 3 mM NAC (Fig. 5B). To further explore the role of ROS, we directly induced ROS production by chemicals (Fig. S4A). Since ATN-224 (ATN) is an inhibitor of superoxide dismutase 1 (SOD1), an abundant cytosolic enzyme that dismutates superoxide into hydrogen peroxide and molecular oxygen [43], we thus utilized ATN in endoderm differentiation. We found ATN-treated cells exhibited a greatly decreased CXCR4- and SOX17-positive percentage in a concentration-dependent manner (Fig. 5C), phenocopying the effect of mitochondrial inhibitor treatment. Moreover, levels of *FOXA2* RNAs were significantly decreased upon being treated with 10 μM ATN (Fig. 5D). These data together indicate that excessive ROS due to mitochondrial dysfunction can impair DE differentiation.

Since both ATP and ROS participated in DE differentiation, we supplemented ATP and NAC simultaneously when treated with the chemical against normal mitochondrial function. Again, ATP and NAC could rescue DE differentiation treated with dynasore, respectively. Importantly, the combined addition of both ATP and NAC could fully compensate for the blocking effect of dynasore, even at low concentrations (i.e.,1 mM NAC and 0.5 mM ATP) (Fig. 5E). These data together suggest the importance of mitochondrial function in endodermal differentiation, which depends on the level of ATP and appropriate ROS (Fig. 5F).

### ATP and NAC can reduce the demand of activin A to promote DE differentiation

**Fig. 6:**
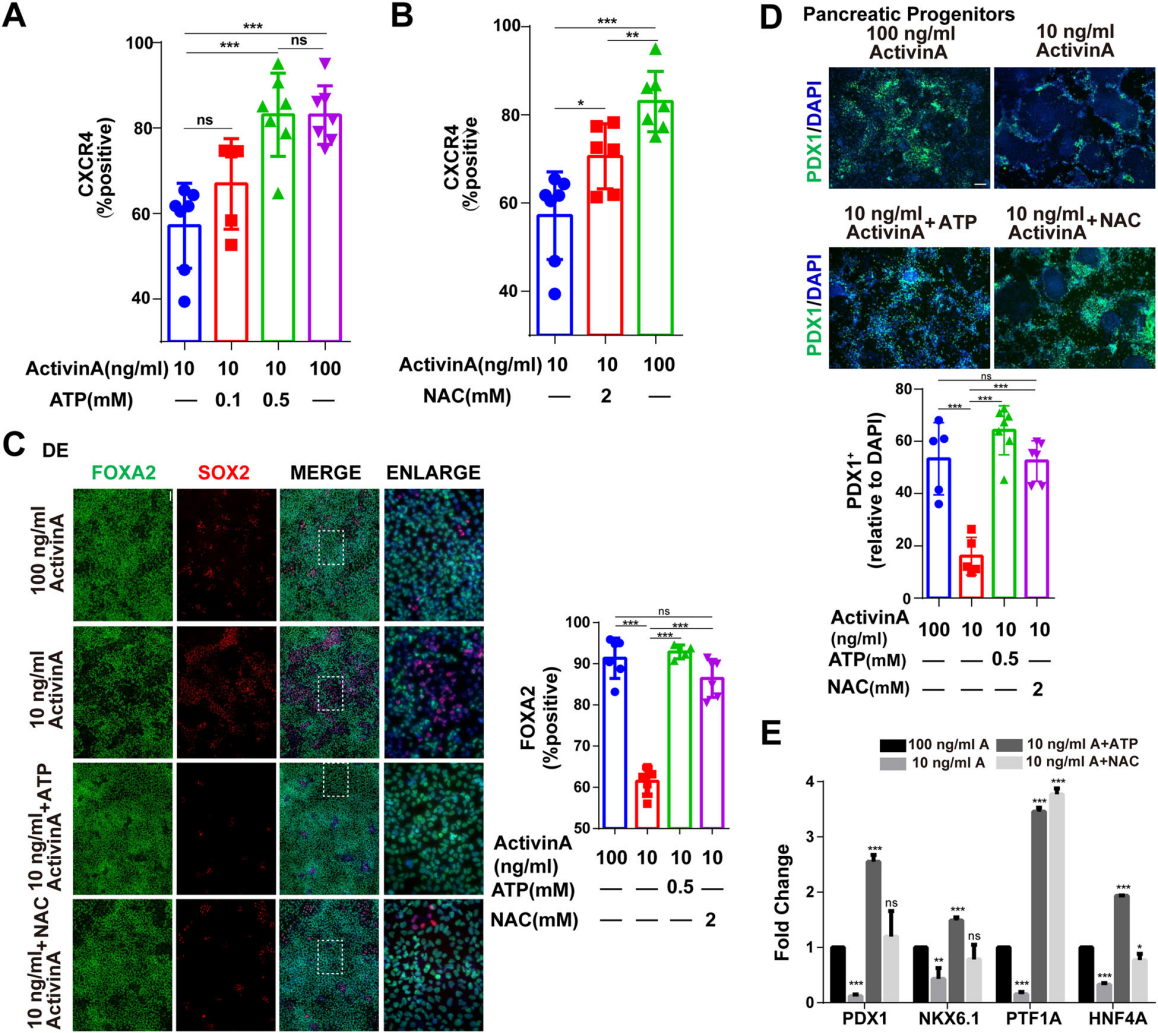
ATP and NAC can reduce the demand of activin A for DE differentiation. **A** Percentage of CXCR4-positive cells (flow cytometry) in DE differentiation with 100 ng/ml or 10 ng/ml Activin A plus different concentrations of ATP (*n* = 7). **B** Percentage of CXCR4-positive cells (flow cytometry) in DE differentiation with 100 or 10 ng/ml Activin A plus 2 mM NAC (*n* = 7). **C** Immunofluorescence analysis of DE cells marked with FOXA2 (green) (nucleus, DAPI, blue; pluripotency, SOX2, red). Quantitative statistics were shown on the right (*n* = 5). Scale bar = 100 μm. **D** Immunofluorescence analysis of pancreatic progenitors marked with PDX1 (green) (nucleus, DAPI, blue). Quantitative statistics were shown on the bottom (*n* = 5). Scale bar = 100 μm. **E** The mRNA expression of representative genes of pancreatic progenitors including *PDX1*, *NKX6.1*, *PTF1A*, and *HNF4A*. Data are normalized to the mRNA level of the 100 ng/ml activin A group (*n* = 3). All data are shown as mean ± SD. ns, not significant. *P < 0.05, **P < 0.01 and ***P < 0.001.

As a member of the TGF-β superfamily and the key factor for the induction of DE differentiation, activin A is applied in a majority of differentiation protocols to generate endoderm and endodermal lineage cells. However, as a growth factor, activin A is at a high cost due to the need for high concentration, which becomes a major concern in cell therapy applications. Recent findings suggest activin A can regulate mitochondria [44], but a high concentration of activin A also induces cellular apoptosis [45]. Thus, we are wondering if ATP or NAC can replace activin A at least partially to achieve efficient DE differentiation. We found neither ATP nor NAC can fully replace activin A; however, we found we could reduce the concentration of activin A from 100 ng/ml (commonly used) to 10 ng/ml when adding ATP or NAC during differentiation. The percentage of CXCR4 positive cells decreased from 80% to 60% after the reduced Activin A concentration. However, 0.5 mM ATP or 2 mM NAC could weaken this reduction, where the ATP group can improve the percentage of CXCR4-positive cells up to 80% (Fig. 6A, B). Immunofluorescence results showed that the percentage of FOXA2-positive cells had a consistent trend with the above data (Fig. 6C). To further verify whether the obtained DE cells could undergo subsequent differentiation, we used the pancreatic lineage differentiation system that we previously reported [46, 47]. Indeed, we were able to obtain comparable PDX1-positive pancreatic progenitor cells compared to the control group (~60%) (Fig. 6D). RT-PCR analysis revealed the upregulation of pancreatic genes like *PDX1*, *NKX6.1*, *PTF1A*, and *HNF4A* in ATP/NAC groups compared to low activin A group and even normal activin A group (control), indicating the potency of the ATP/NAC-derived DE cells (Fig. 6E). In addition, the simultaneous supplementation of 0.1 mM ATP and 2 mM NAC could achieve comparable differentiation efficiency with 100 ng/ml activin A group (> 80%), while 0.1 mM ATP had little effect on DE when added alone (Fig. S6A). These data together show that ATP and NAC can serve as new supplemental ingredients to replace parts of activin A for endodermal differentiation, likely reducing the cost of regenerative medicine and with valuable application potential.

## Discussion

Mitochondria provide the necessary energy for various life activities and play a vital role in development, tumorigenesis, immune regulation, and other biological processes [48–51]. Here, we investigated the role of mitochondria during the DE differentiation of human PSCs. Our results showed the mitochondria in DE cells are more mature and active than that in undifferentiated PSCs. The interference of mitochondrial function including biogenesis, fission, and ATP production by either gene editing or chemical targeting can hinder DE differentiation, suggesting that normal mitochondrial function is essential for DE differentiation. In addition, this interference can be alleviated by ATP addition or ROS scavenger. Moreover, the combined treatment of ATP and NAC can further rescue the DE differentiation defect due to mitochondrial dysfunction. These data together demonstrate that mitochondria play a critical role in DE differentiation, which is mainly regulated by ATP and ROS.

In recent years, scientists are more and more interested in mitochondrial function in diverse biological processes. Multiple studies showed that undifferentiated ESCs [52, 53] or iPSCs [54, 55] have un-fused spherical mitochondria with poorly developed cristae and perinuclear distribution compared to their long, branched, and cristae-rich somatic cell counterparts dispersed in the cytoplasm. The immature mitochondrial features and distribution similar to stem cells can be also observed in early embryos [56]. Meanwhile, the metabolic profile of differentiated cells derived from stem cells shifts from glycolysis to OXPHOS corresponding to mitochondrial remodeling [55]. However, there are some exceptions. For instance, mesenchymal stem cells have a relatively more tubular shape of mitochondria but with glycolysis-dependent energy metabolism [57]. Thus, the morphology, distribution, and function of mitochondria are heterogeneous in different cell types and environments. Functionally, one group knocked out multiple mitochondrial fission genes in ESCs separately, demonstrating that the impaired mitochondrial homeostasis does not disrupt ESC pluripotency but alters the metabolic phenotype and ATP production [58]. In addition, mitochondrial homeostasis-related factors also play a crucial role in embryonic development [59] and the differentiation process, including neuronal [60], myogenic [61], cardiac [62], and mesenchymal stem cell differentiation [13]. These studies imply that mitochondria may have different regulatory roles in the different stages of development. Here, we have revealed that mitochondria actively participate in human DE differentiation. We noticed that Ma and colleagues showed that CHIR-99021 could promote mitochondrial biogenesis and function via β-catenin signaling pathway and miRNA expression during human definitive endodermal progenitor cells differentiation, but this study was performed by using human adipose stem cells [26].

Mitochondria play an important role in metabolism; however, how mitochondria function as a basic metabolic organelle is still not very clear. The main functions of mitochondria are to accomplish oxidative phosphorylation and produce ATP and ROS [63]. In our study, we showed ATP and ROS contributed to DE differentiation as the major downstream of mitochondrial function. However, how ATP and ROS further regulate endoderm differentiation remains to be studied. The phosphorylation modification of proteins requires ATP to provide phosphoric acid and is catalyzed by protein kinases [64]. As a result, changes in ATP levels may affect the phosphorylation of some key proteins and in turn, regulate endoderm differentiation. Normal physiological production of ROS can function as a second messenger to activate the JAK/STAT and nuclear factor of activated T cells (NFAT), but excessive ROS production has the opposite effect [65, 66]. This dose-dependent bidirectional regulatory effect is interesting, but the mechanism is not fully understood. In addition, growing studies show that ROS is involved in the epigenetic process in cellular activities through DNA methylation and histone modification [67]. For example, hydrogen peroxide caused the upregulation of DNA methyltransferase 1 (DNMT1) and histone deacetylase 1 (HDAC1) [68], and could increase histone H4 acetylation and HAT activation as well [69]. We would explore the underlying mechanism of ATP or ROS regulating human DE differentiation in the following studies.

Given the high cost of activin A, attempts have been made to replace activin A with small molecules. A recent publication from Jiang et al. [70] showed that low-level WNT activation and BMP inhibition by small molecules only could yield DE cells. However, this small-molecule approach depends on endogenous activin/Nodal signaling, thereby reducing its universality. In addition, a recent study showed that the high dose of activin A (100 ng/ml) causes higher cell death in endoderm differentiation [71]. Here, we reported that a low concentration of activin A (10 ng/ml) together with ATP/NAC was able to efficiently obtain DE cells which maintained the ability to continue backward differentiation.

In conclusion, our present study has revealed mitochondrial remodeling during human DE differentiation. More importantly, we have demonstrated by genetic and chemical approaches that mitochondria play a crucial role in DE differentiation, which is mainly due to ATP production as ROS might play an inhibitory role. Meanwhile, this mechanism can be used to optimize the DE differentiation scheme with a decreased demand for activin A. Our findings extend the importance of mitochondria in developmental biology and regenerative medicine, providing new insights into the mechanism of cell fate determination.

## Materials and methods

### Cell culture and differentiation

Undifferentiated human ESC HUES8 and iPSC PGP1 were maintained in mTeSR1 (StemCell Technologies, Cat# 85850) on Matrigel-coated cell culture plates at 37 °C with 5% CO_2_. For directed differentiation of DE, human PSCs were cultured in DMEM-F12 or DMEM medium supplemented with 0.2% bovine serum albumin (Yeasen Biotechnology, Cat# 36101ES76), 100 ng/mL Activin A (PeproTech, Cat# 120-14P), 2.5 μM CHIR99021 (Selleck, Cat# S2924) for 24 h, then the CHIR99021 was removed from a medium for the following 3–4 days. Next, the differentiation of the pancreatic progenitor cells was performed according to a previous report [47]. Small molecules used in this study are listed as follows: XCT790 (XCT), 2–5 μM (MCE, Cat# HY-10426); ATN-224 (ATN), 5–10 μM (MCE, Cat# HY-16074); Mdivi-1, 10 μM (MCE, Cat# HY-15886); IACS-010759 (IACS), 0.1–20 nM (Selleck, Cat# S8731); Dynasore (DYNA), 1–3 μM (Selleck, Cat# S8047); FCCP, 1–2 μM (Selleck, Cat# S8276); Oligomycin A (Oligo), 0.1–10 μM (Apexbio, Cat# A5588); Doxycycline (DOX), 100 ng/ml (Sigma, Cat# 324385). Of note, the concentrations of the chemicals we used in this study showed little effect on the survival of cells.

### Transmission electron microscopy

HUES8 cells were cultured on a 10-cm dish and differentiated as indicated above. The cell pellet was obtained by centrifugation after suspension. Then the pre-cooled 2.5% glutaraldehyde (1–2 ml/sample) was slowly added to the cell pellet along the tube wall. After 1 h of fixation (room temperature and light avoidance), the samples were sent to the facility of the School of Basic Medical Sciences, Wuhan University. Finally, the sample was sliced by Servicebio (Wuhan) and photographed under the transmission electron microscope (School of Basic Medical Sciences, Wuhan University). Mitochondrial images were analyzed using ImageJ.

### Immunofluorescence staining

Cells on tissue culture plates were fixed in 4% paraformaldehyde for 15 min, washed using phosphate-buffered saline (PBS) 3 times, and then blocked and permeabilized with 10% donkey serum and 0.3% Triton X-100 in PBS for 1 h at room temperature. Primary antibodies (in 1× PBS with 0.3% Triton X-100 and 10% donkey serum) were incubated overnight at 4 °C. The next day, the cells were washed three times with PBS and incubated with secondary antibodies (in 1× PBS with 0.3% Triton X-100 and 10% donkey serum) for 1–2 h at room temperature. Finally, cells were washed three times using PBS and stained with DAPI for 10 min at room temperature. The primary antibodies used in this study include mouse anti-OCT4 (Santa Cruz, Cat# SC-5279), mouse anti-SOX2 (BD Biosciences, Cat# 561469), rabbit anti-FOXA2 (HUABIO, China, Cat# ET1703-76), and goat anti-SOX17 (R&D, Cat# AF1924).

### Flow cytometry

Cells were washed with PBS, detached by TrypLE, and then resuspended in 2% fetal bovine serum. APC-conjugated antibody against CXCR4 (1:200, BD Biosciences, Cat# 555976) was added for 30 min at 4 °C. Then, FITC-conjugated antibodies against intracellular marker SOX17 (1:200, BD Biosciences, Cat# 562205) were stained using The BD PharmingenTM Transcription Factor Buffer Set (BD Biosciences, Cat# 562574) according to the manufacturer’s instructions. The resulting cells were analyzed on the BD LSRFortessaX-20. The FACS data were processed by FlowJo.

### Cell lysis and immunoblotting

Cells were harvested by digestion with Trypsin. After centrifugation at 1000 rpm, cells were resuspended in ice-cold lysis buffer (Beyotime Biotechnology, Cat# P0013C) for 30 min on ice. Lysates were centrifugated at 10,000*g* for 10 min and the supernatant was collected. Western blot analysis was performed using standard protocols, and the following commercial antibodies were used: rabbit anti-TFAM (Abcam, Cat# 176558), rabbit anti-beta Actin (Proteintech, Cat# 20536-1-AP), rabbit anti-TOMM20 (Abclonal, Cat# A6774), and rabbit anti-TIMM23 (Abclonal, Cat# A8688), mouse anti-α-Tubulin (Abclonal, Cat# AC012).

### Detection of Intracellular ROS

PSCs or DE cells were seeded into the glass-bottomed slides. On the day of measurement, the culture medium was removed and cells were washed with PBS three times. Then cells were incubated with CellROX Green Reagent (Invitrogen, Cat# C10444) at 37 °C for 20 min. Meanwhile, the nucleus was incubated with Hoechst. The images were captured using Zeiss LSM 880 and analyzed by ImageJ. Both types of cells could also be digested and then incubated for 20 min at 37 °C with CellROX. Subsequently, the incubated cells were analyzed on ACEA NovoCyte. When detecting ROS levels using FACS, we gated the experimental samples using dye-free and H_2_O_2_-treated cells as negative and positive controls, respectively.

### Mitochondrial DNA determination

Total DNA was purified from undifferentiated human ESCs (HUES8) and DE cells using the HiPure Tissue DNA Mini Kit (Magen, Cat# D3121-02). mtDNA was detected through the mitochondrial genome encoded gene ND1 by quantitative PCR (qPCR), with the nuclear-encoded gene GAPDH as a reference. The quantitative PCR (qPCR) reactions were performed with 2×SYBR Green qPCR Master Mix (Bimake, Cat# B21203) on 384-well Bio-Rad CFX384. Primers used are as follows:

ND1: F- CCCTAAAACCCGCCACATCT

ND1: R-GAGCGATGGTGAGAGCTAAGGT

GAPDH: F- AAGTGGATATTGTTGCCATC

GAPDH: R-GGAATACGTGAGGGTATGAA

### Mitochondria imaging

For mitochondria imaging, cells cultured in a 35-mm^2^ glass-bottom dish were stained with MitoTracker Red CMXRos (Invitrogen, Cat# M7512), which was carried out according to the manufacturer’s protocol. Then, mitochondria were imaged using Zeiss LSM 880 confocal microscope. The mitochondrial average length was analyzed by a tool, MINA [19].

### XF24 extracellular flux

PSCs or DE cells were seeded into the cell culture microplate and incubated at 37 °C incubators with 5% CO_2_ overnight. The DMEM assay medium (XF assay-modified DMEM supplemented with 10 mM XF glucose, 1 mM XF pyruvate, 2 mM XF glutamine, pH 7.4) was prewarmed at 37 °C to use. Then the OCR and ECAR measurement were assessed by Seahorse XF Cell Mito Stress Test Kit (Agilent, Cat# 103015-100) and Seahorse XF Glycolysis Stress Test Kit (Agilent, Cat# 103020-100), respectively. Here, 1.5 µM oligomycin (Oligo), 0.5 µM FCCP, and 0.5 µM rotenone/antimycin A (Rot/AA) were used in Mito Stress Test assay. 10 mM glucose, 1 µM oligomycin, 50 mM 2DG were used in the Glycolysis Stress Test assay. Finally, the cell number was normalized using CyQUANT™ Cell Proliferation Assay Kit (Invitrogen, Cat# C7026) or Pierce^™^ BCA Protein Assay Kit (Thermo Fisher, Cat# 23225).

### Measurement of lactate production

Lactate production was measured using a Lactic Acid assay kit (NanJing Jiancheng Bioengineering Institute, Cat# A019-2) according to the manufacturer’s protocol. Absorbance was measured by the SpectraMax i3× microplate-reader (Molecular Devices) at 530 nm. The cells were normalized with DNA content using the CyQUANT^™^ Cell Proliferation Assay Kit (Invitrogen, Cat# C7026).

### Luminescence ATP determination

ATP/ADP ratio was measured using the bioluminescent-based ADP/ATP Ratio Assay Kit (Abcam, Cat# ab65313) according to the manufacturer’s protocol. In brief, the cells of different states or treatments were replated into a new Matrigel-coated 96-well plate at the indicated density (i.e., 10^3^–10^4^ cells the day before ATP/ADP was detected). The luminescence intensity was measured on the SpectraMax i3× microplate-reader (Molecular Devices) with luminescence read mode. Finally, the cells were normalized with DNA content using the CyQUANT^™^ Cell Proliferation Assay Kit (Invitrogen, Cat# C7026).

### ATP and NAC replenishment assay

ATP (Sigma, Cat# A7699) was dissolved in water at 80 mM and adjusted to pH7.0. ATP storage solution was added directly to the medium to obtain DE differentiation medium with different final concentrations of ATP (0.5–2 mM) for 3–4 days.

NAC (Sigma, Cat# A9165) was dissolved in water at 600 mM. And the method of use (1–3 mM) is the same as above ATP.

### RT-PCR

Total RNA was extracted using the HiPure Total RNA Mini Kit (Magen, Cat# R4111-03). Then, 1 μg of total RNA was reverse-transcribed into cDNA by ABScript II RT Master Mix for qPCR (ABclonal, Cat# RK20402). The cDNA was used for quantitative polymerase chain reaction with 2×SYBR Green qPCR Master Mix (Bimake, Cat# B21203). The qRT-PCR was performed on the BioRad CFX384 RT-PCR System. GAPDH was used as the internal reference for mRNA and the 2^−^^ΔΔCt^ method was used to analyze the mRNA relative expression. Primers used are as follows:

*OCT4*: F- CAAAGCAGAAACCCTCGTGC; R- TCTCACTCGGTTCTCGATACTG;

*SOX2*: F- GTCATTTGCTGTGGGTGATG; R- AGAAAAACGAGGGAAATGGG;

*NANOG*: F- CCCCAGCCTTTACTCTTCCTA; R- CCAGGTTGAATTGTTCCAGGTC;

*FOXA2*: F- GGAGCAGCTACTATGCAGAGC; R- CGTGTTCATGCCGTTCATCC;

*SOX17*: F- GCATGACTCCGGTGTGAATCT; R- TCACACGTCAGGATAGTTGCAGT;

*CXCR4*: F- TACACCGAGGAAATGGGCTCA; R- AGATGATGGAGTAGATGGTGGG;

*PDX1*: F- TTAGGATGTGGACGTAATTCCTGTT; R- GGCCACTGTGCTTGTCTTCA;

*NKX6.1*: F- AGACCCACTTTTTCCGGACA; R- CCAACGAATAGGCCAAACGA;

*PTF1A*: F- CAGGACACTCTCTCTCATGGA; R- TGGTGGTTCGTTTTCTATGTTGT;

*HNF4A*: F- ACTACATCAACGACCGCCAGT; R- ATCTGCTCGATCATCTGCCAG;

*GAPDH*: F- AATGAAGGGGTCATTGATGG; R- AAGGTGAAGGTCGGAGTCAA;

### Untargeted metabolite analysis

The cells of a different state (HUES8 cell line) were cultured on a 10-cm dish. Every type of sample had seven replicates. The cells were washed and then liquid nitrogen was directly added to the dish on dry ice. After lysis at −80 °C refrigerator for 20 min, the cells in the dish were scraped with precooled 80% methanol and operated on dry ice. Finally, the supernatant was collected by centrifugation and tested in Dr. Tiangang Liu Laboratory of School of Pharmacy, Wuhan University. Ultrahigh liquid chromatography equipped with Q Exactive benchtop Orbitrap mass spectrometer system was used for untargeted metabolite analysis. Hydrophilic interaction chromatography and reverse-phase chromatography were both used with two mass spectrum ion modes: positive mode and negative mode. Cells from ESC, intermediate samples, and DE cells were collected for metabolite analysis. The obtained liquid chromatography-mass spectrometry data were processed using Compound Discoverer 3.0 (Thermo Scientific).

### RNA sequencing and data analysis

RNA samples extracted from the precipitate after extraction of metabolites were sent to Annoroad (China) for sequencing. The quality control of all obtained reads was accessed with FastQC (version 0.11.8) and the reads were aligned against the reference human genome (hg38) with HISAT2 (version 2.1.1). Raw gene expression (raw reads count) was calculated by featureCounts (version 1.6.4). Expression values were accessed in units of transcripts per million mapped reads from the raw reads count. Genes with fold change ≥ 2 were defined as DEGs. Enrichment analysis of gene ontology and KEGG pathway were performed using clusterProfiler package in R. The Gene Expression Omnibus (GEO) accession number for the RNA-seq raw data reported in this work is GSE168625. Gene expression profiling of pancreatic differentiation from hPSCs used in this study is from GSE114099 [21].

### Quantification and statistical analysis

In this study, data were represented as mean values with error bars indicating standard deviations (±SD), which were calculated from three independent experiments at least. *P* < 0.05 was considered as statistical significance. Data were analyzed and visualized using GraphPad Prism or R software. *P < 0.05, **P < 0.01, ***P < 0.001 using Student’s *t*-test (two-tailed, equal variance).

## Supplementary information

Supplementary figure legends

Figure S1

Figure S2

Figure S3

Figure S4

Figure S5

Figure S6

Original Data

## Data Availability

The data generated or analyzed during this study are included in this published article and its Supplementary Information files.
