# Supplementary material for: Mitochondrial homeostasis regulates definitive endoderm differentiation of human pluripotent stem cells
Source: Cell Death Discov. 2022 Feb 17;8:69. doi: 10.1038/s41420-022-00867-z (PMC8854419; doi:10.1038/s41420-022-00867-z)
Supplement: Supplementary file 1 — Supplementary figure legends [file 41420_2022_867_MOESM1_ESM.docx]

*Supplementary information*

**Mitochondrial homeostasis regulates definitive endoderm differentiation of human pluripotent stem cells**

Jing Lv^1,#^, Ying Yi^1,#^, Yan Qi^2^, Chenchao Yan^1^, Wenwen Jin^1^, Liming Meng^1^, Donghui Zhang^2,^*, Wei Jiang^1,3,^*

^1^ Department of Biological Repositories, Frontier Science Center for Immunology and Metabolism, Medical Research Institute, Zhongnan Hospital of Wuhan University, Wuhan University, Wuhan 430071, China

^2^ State Key Laboratory of Biocatalysis and Enzyme Engineering, School of Life Science, Hubei University, Wuhan, 430062, China;

^3^ Human Genetics Resource Preservation Center of Wuhan University, Wuhan 430071, China;

^#^ These authors contribute equally.

* To whom correspondence should be addressed.

Wei Jiang: 116 East-Lake Road, District of Wuchang, Medical School of Wuhan University, Wuhan, Hubei Province 430071, People’s Republic of China. Telephone: 86-27-68750399; e-mail: jiangw.mri@whu.edu.cn, jiangw.pku@gmail.com;

Donghui Zhang: 368 Youyi Road, District of Wuchang, School of Life Science, Hubei University, Wuhan, Hubei Province 430062, People’s Republic of China. Telephone: 86-27-88663882; e-mail: dongh.zhang@hubu.edu.cn

**Figure S1. Evaluation of DE differentiation system and measurement of mitochondrial morphological changes.**

1. The differentially expressed genes in early (D2, 2 days’ differentiation) and DE cells relative to PSCs. Dot plot of gene expression profiling was generated using normalized expression values in log2 (TPM+0.1). Differentially expressed genes (DEGs) during differentiation was highlighted in red (up regulation) or blue (down regulation). Genes with fold-change≥2 was defined as DEGs after eliminating genes with low expression level (TPM≤2).
2. Enriched GO terms of DEGs in the category of Biological Process.
3. KEGG pathway analysis of DEGs for early differentiation and DE cells relative to ESCs.
4. Quantitative statistics of mitochondrial average length with MitoTracker staining at different stages of DE differentiation from ESCs (n=6).
5. Overlapping of ES/DE DEGs and mitochondria related genes in RNA-seq analysis.
6. The chord diagram presents the overlap between the differentially expressed genes after DE differentiation of ESCs.
7. Heatmap showing the expression of mitochondrial homeostasis related genes by RNA-seq.

All data are shown as mean ± SD. *** P<0.001.

**Figure S2. The remodeling of mitochondrial function in DE differentiation.**

1. Measurement of intracellular ROS level by confocal during DE differentiation of iPSCs. Quantitative statistics of the relative intensity of the ROS level was shown on the right. Scale bar=20μm.
2. Quantitative statistics the mean fluorescence intensity (MFI) of the ROS level during DE differentiation of iPSC by FACS (n=3). Neg, negative control.
3. Extracelluar Acidification Rate (ECAR) from Seahorse assay following pharmacological modifications (n=3). Oligo, oligomycin.
4. Seahorse Mito Stress Kit assay on ESCs and DE cells (n = 3). Rot/AA, rotenone/antimycin A.
5. The mRNA expression of succinate-CoA ligase (SUCL) by RNA-seq.

All data are shown as mean ± SD. ns, not significant. * P<0.05, ** P<0.01 and *** P<0.001.

**Figure S3. Heterozygous knockout of TFAM impairs DE differentiation.**

1. The mtDNA expression relative to the nuclear gene GAPDH in wild-type and TFAM^+/-^ iPSCs (n=3).
2. Basal and maximal respiration in wild-type and TFAM^+/-^ iPSCs by Seahorse Mito Stress Kit assay (n=3).
3. Immunofluorescence analysis of the DE differentiation efficiency with FOXA2 (green) (nucleus, DAPI, blue; pluripotency, SOX2, red) in wild-type and TFAM^+/-^ iPSCs. Scale bar=100μm.

All data are shown as mean ± SD. * P<0.05, ** P<0.01.

**Figure S4.** **Interference with mitochondrial homeostasis impairs DE** **differentiation.**

1. Schematic illustration of inhibitors of mitochondrial homeostasis.

(B-C) Effect of XCT790 (XCT) on the percentage of DE cells from ESCs measured by flow cytometry (B), immunofluorescence (C). Scale bar=400μm.

(D) Flow cytometric analysis to measure the percentage of cells expressing both DE markers (SOX17 and CXCR4) from ESCs treated with dynasore.

1. Flow cytometric analysis to measure the percentage of CXCR4- and SOX17-positive cells in DE differentiation of iPSCs treated with dynasore (n=3).
2. Flow cytometric analysis to determine the percentage of CXCR4- and SOX17-positive cells in DE differentiation of ESCs treated with 10μM MDIVI-1 (n=3).
3. Bright field images of DE cells treated with MDIVI-1 at different concentrations showing no obvious cell death under 10μM. Scale bar=400μm.

All data are shown as mean ± SD. ***P < 0.001.

**Figure S5. Interference with ETC impairs DE differentiation.**

1. MitoTracker staining of DE cells treated with mitochondrial inhibitors, MDIVI-1 or dynasore. Arrows indicate the mitochondrial tubular morphology. Mitochondria of DE cells treated with mitochondrial inhibitors (Inhibition)show larger tubes (abnormal swollen tubes) (right, an illustration of mitochondrial morphology). Scale bar=10μm.
2. Schematic illustration of inhibitors on respiratory transmission chains.
3. Flow cytometric analysis to measure the percentage of CXCR4- and SOX17-positive cells in DE differentiation of iPSCs treated with FCCP (n = 3). All data are shown as mean ± SD. *** P < 0.001.

**Figure S6. ATP and NAC can reduce the demand of activin A to promote DE differentiation.**

1. Percentage of CXCR4-positive cells (flow cytometry) in DE differentiation of ESCs with 100ng/ml or 10ng/ml Activin A plus 0.1 mM ATP and 2mM NAC (n=7). All data are shown as mean ± SD. ns, not significant. * P<0.05, ** P<0.01 and *** P<0.001.
