# Supplementary figures and images for: Mitochondrial homeostasis regulates definitive endoderm differentiation of human pluripotent stem cells
Source: Cell Death Discov. 2022 Feb 17;8:69. doi: 10.1038/s41420-022-00867-z (PMC8854419; doi:10.1038/s41420-022-00867-z)

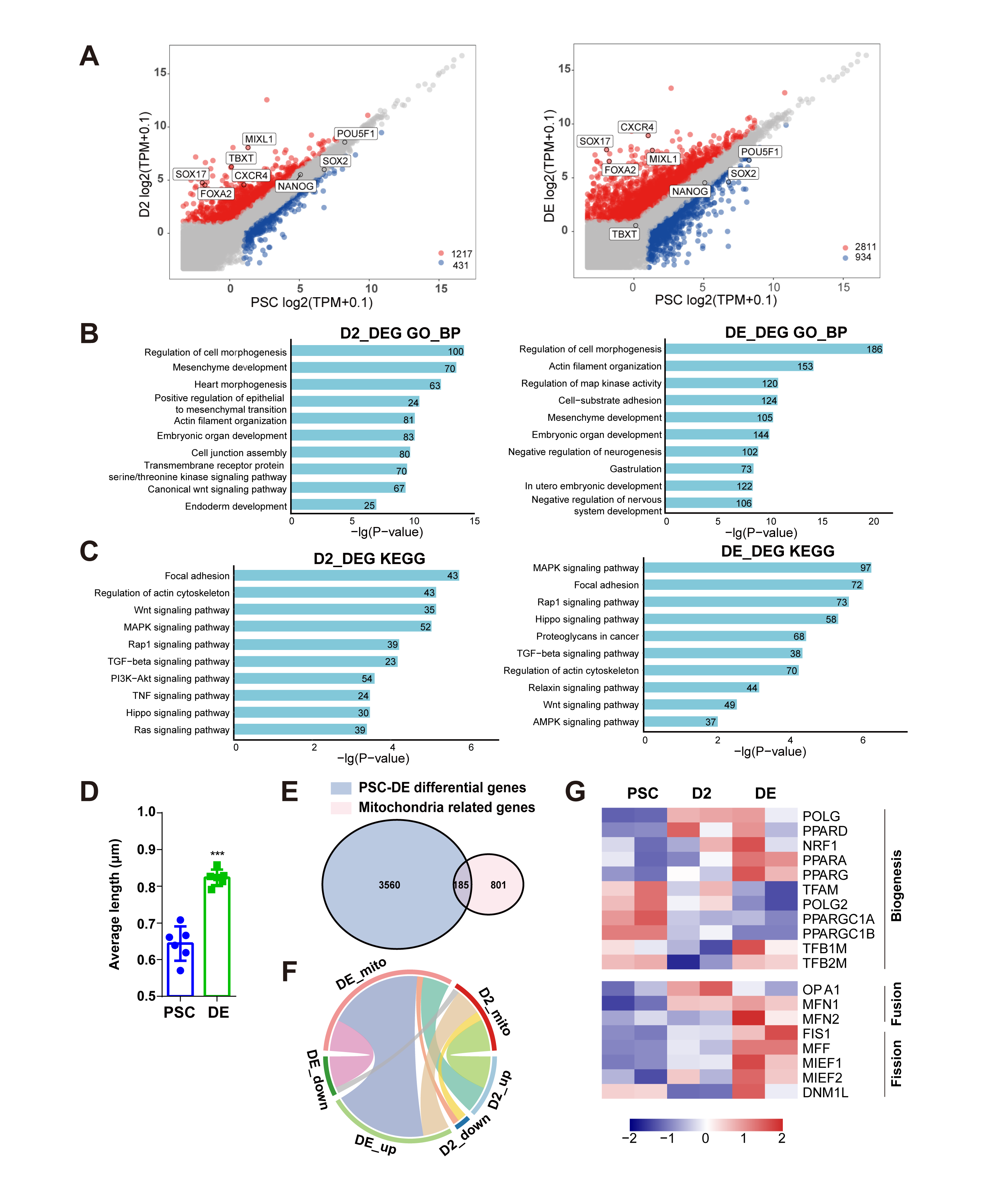

Supplement: Supplementary file 2 — Figure S1 [file 41420_2022_867_MOESM2_ESM.tif]

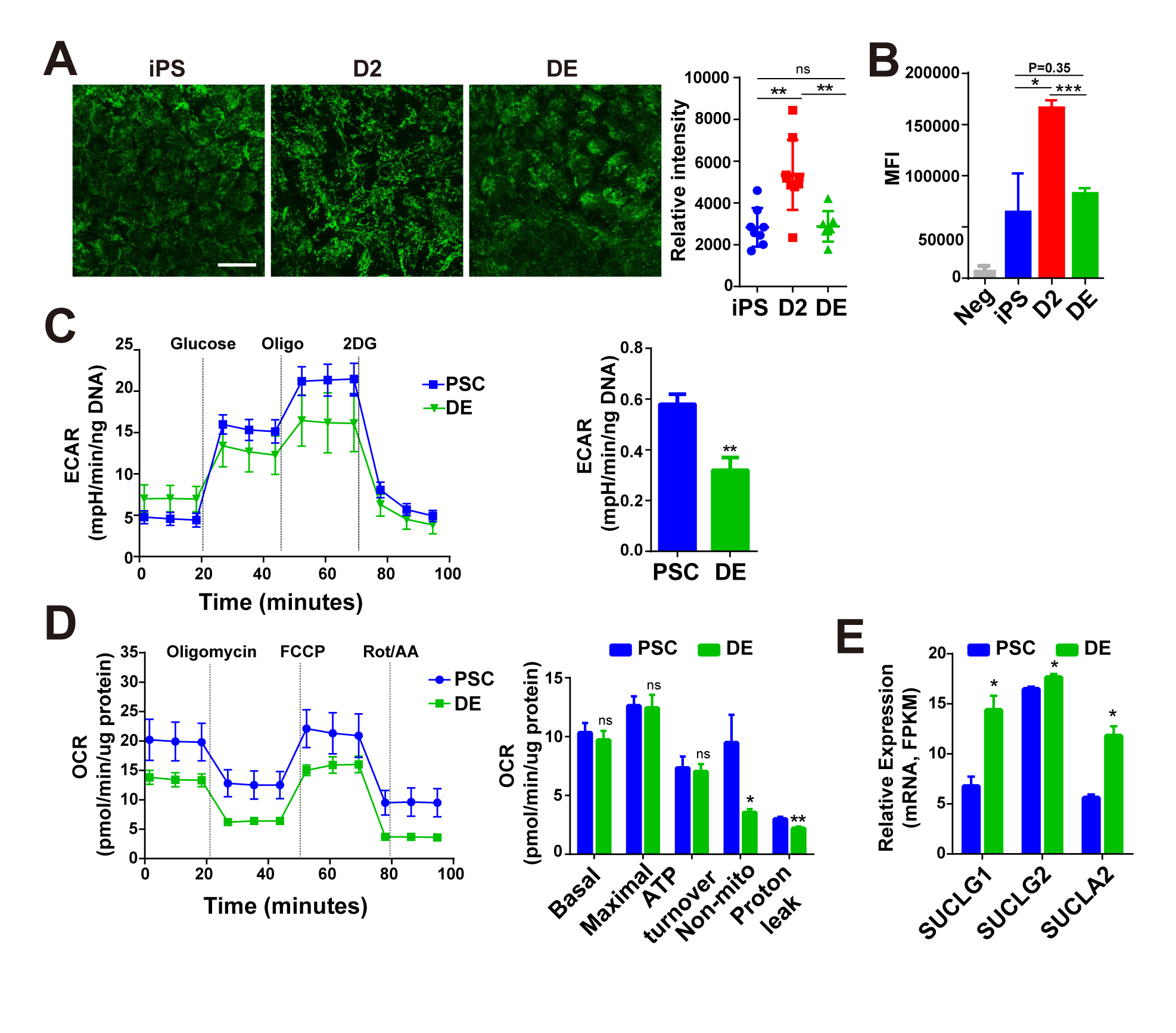

Supplement: Supplementary file 3 — Figure S2 [file 41420_2022_867_MOESM3_ESM.tif]

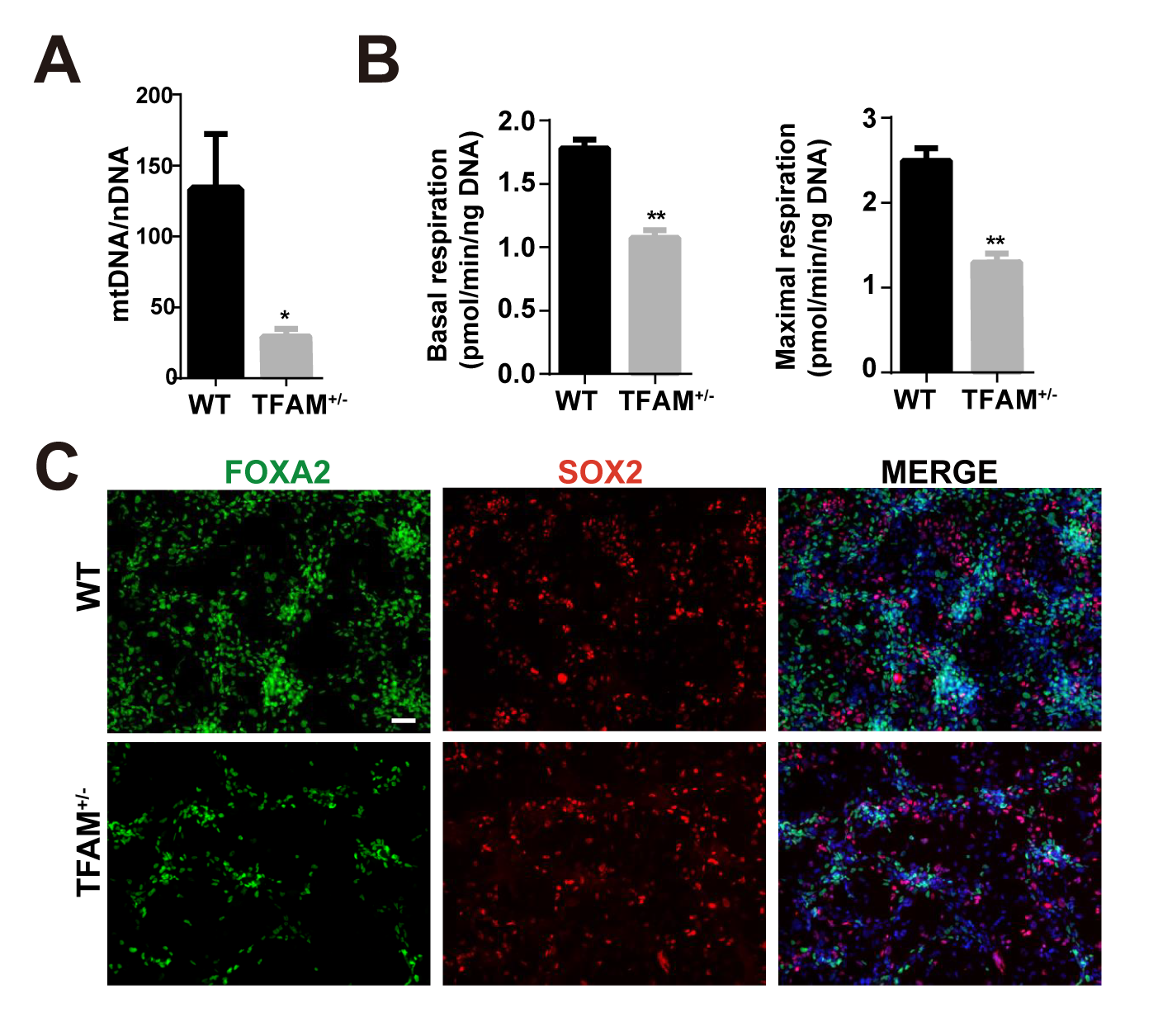

Supplement: Supplementary file 4 — Figure S3 [file 41420_2022_867_MOESM4_ESM.tif]

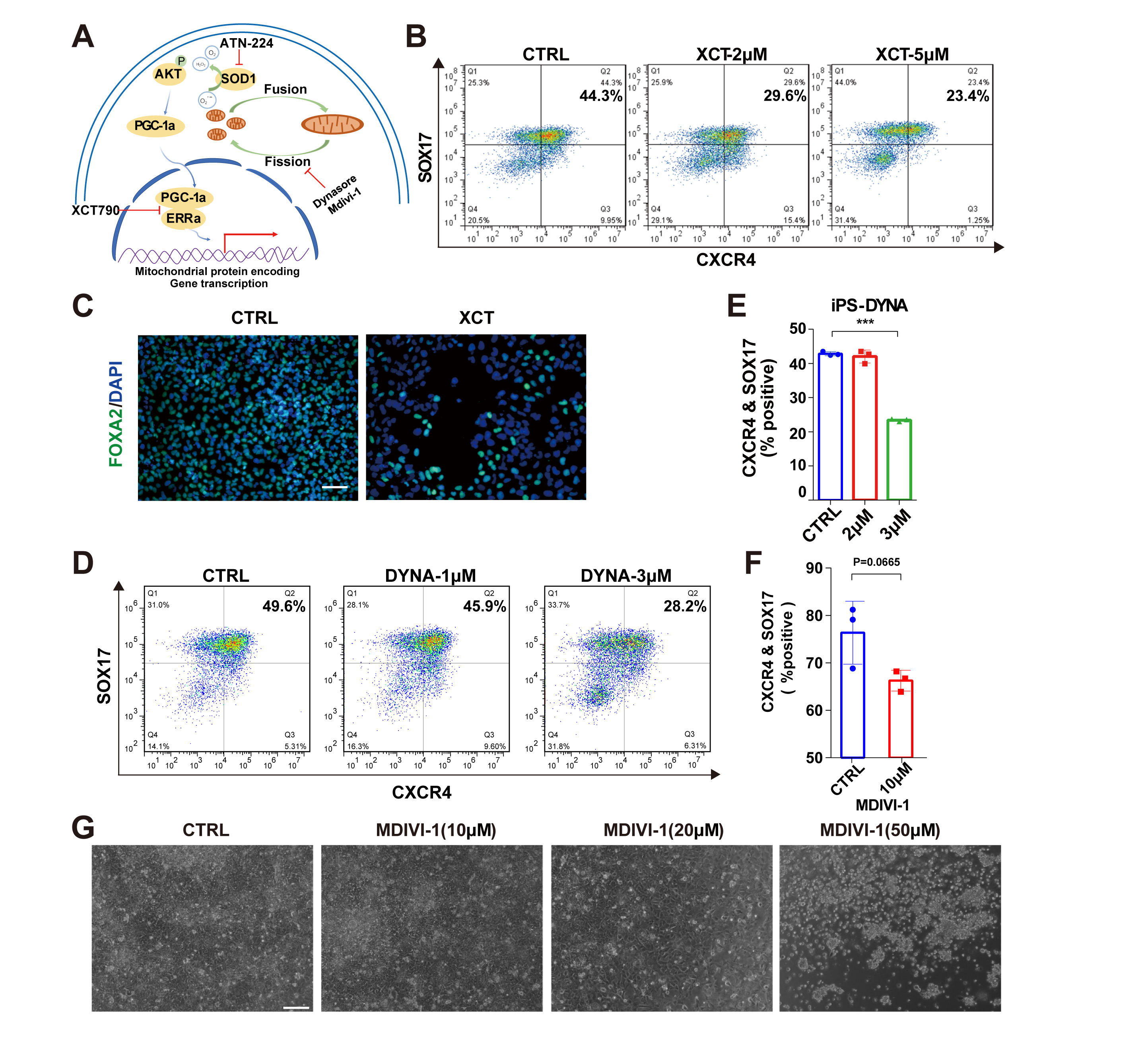

Supplement: Supplementary file 5 — Figure S4 [file 41420_2022_867_MOESM5_ESM.tif]

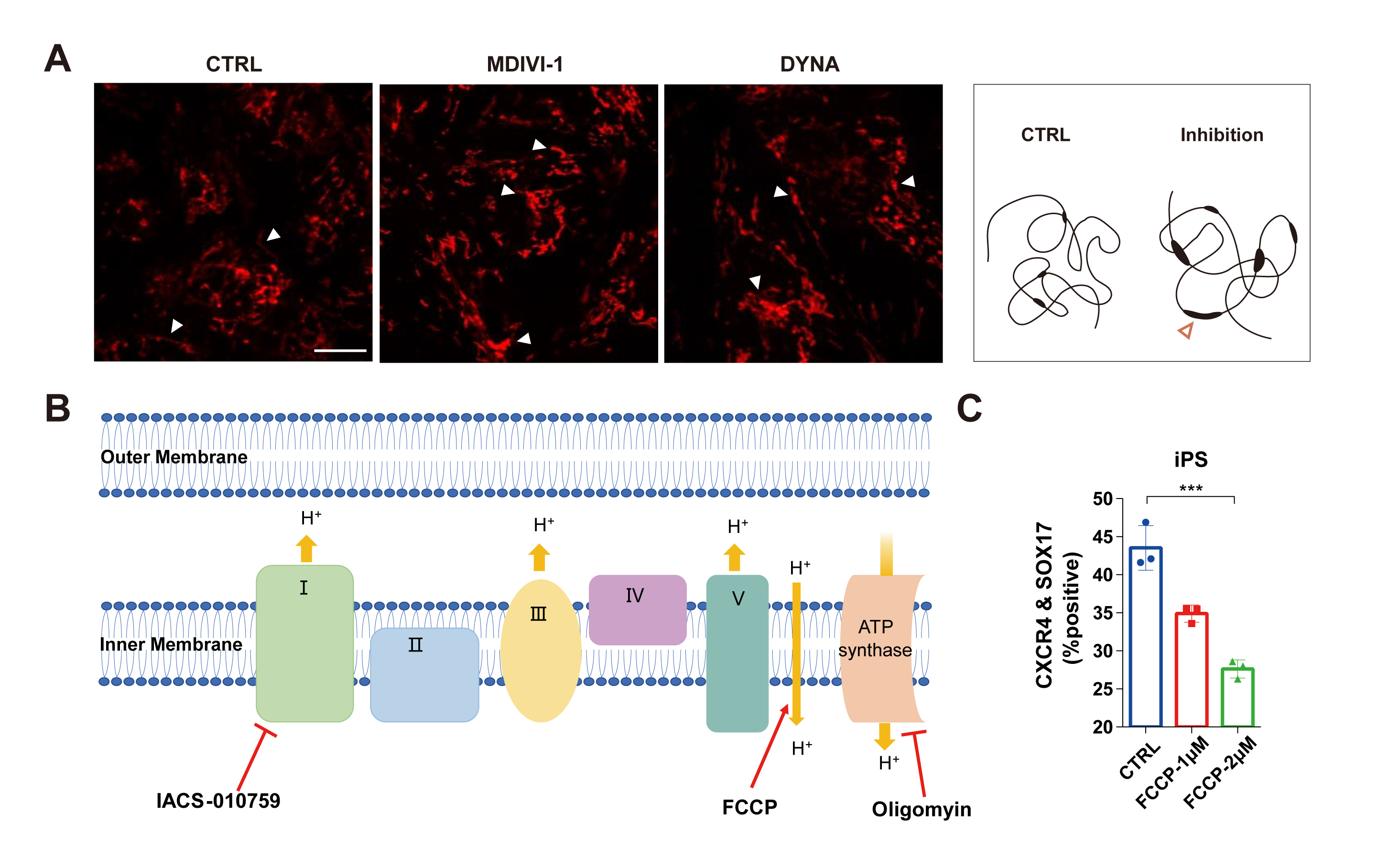

Supplement: Supplementary file 6 — Figure S5 [file 41420_2022_867_MOESM6_ESM.tif]

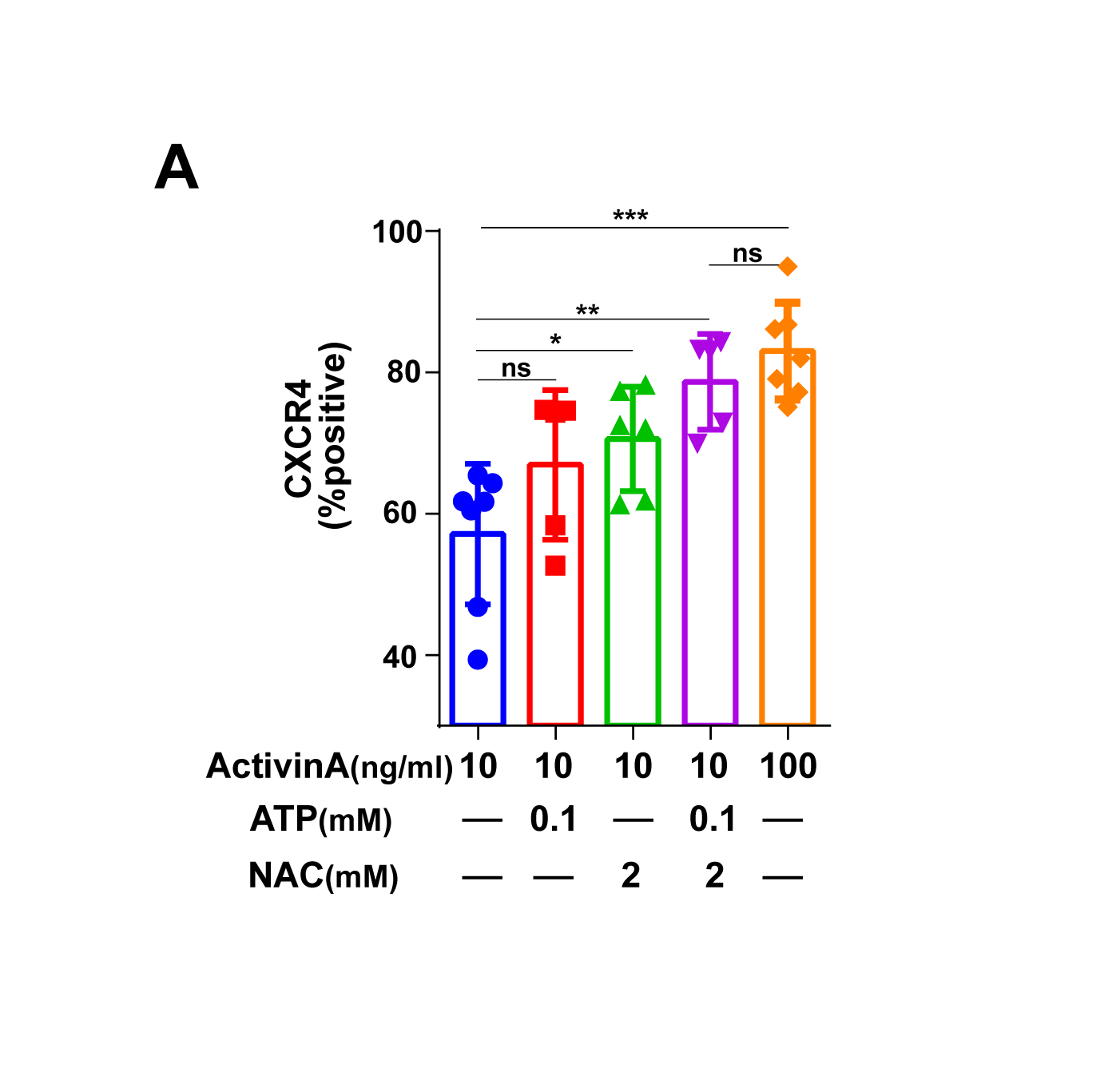

Supplement: Supplementary file 7 — Figure S6 [file 41420_2022_867_MOESM7_ESM.tif]

Figure 2B:

## B

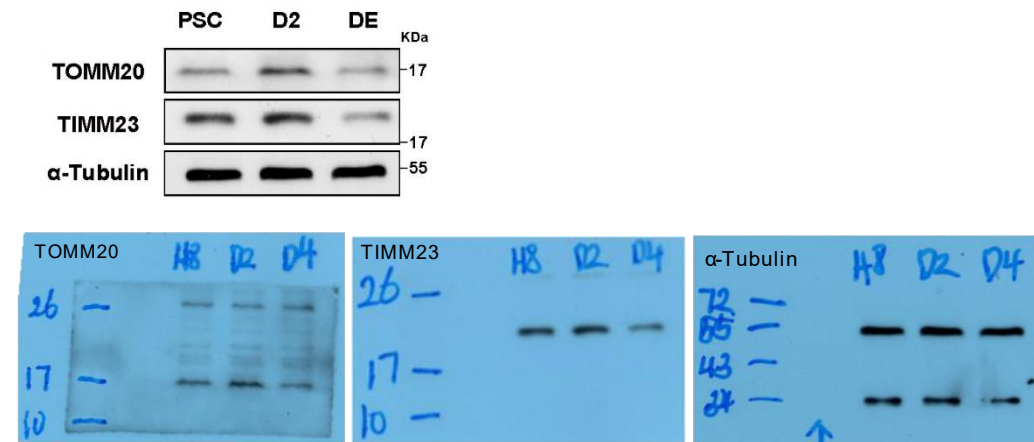

Figure 3A:

**A**

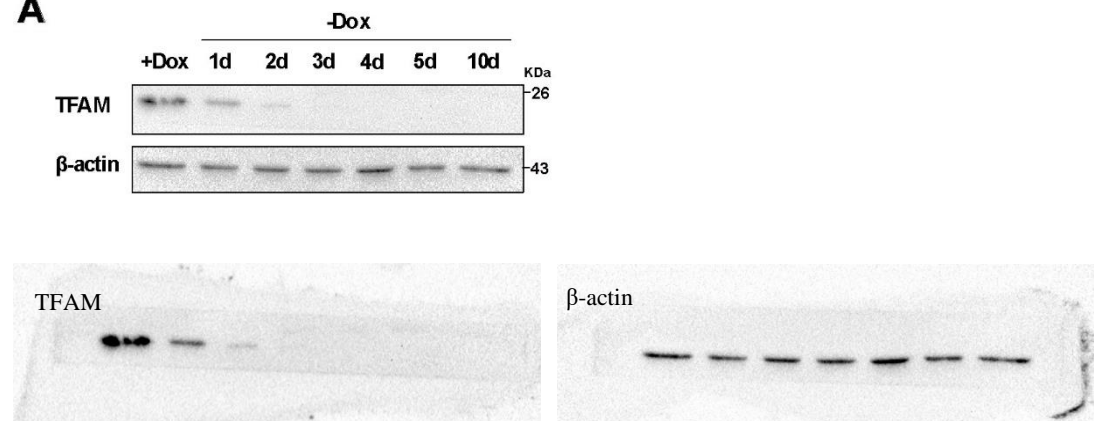

Supplement: Supplementary file 8 — Original Data [file 41420_2022_867_MOESM8_ESM.pdf]
